# Diagnosis and treatment of cardiac amyloidosis: an interdisciplinary consensus statement

**DOI:** 10.1007/s00508-020-01781-z

**Published:** 2020-12-03

**Authors:** Diana Bonderman, Gerhard Pölzl, Klemens Ablasser, Hermine Agis, Stefan Aschauer, Michaela Auer-Grumbach, Christina Binder, Jakob Dörler, Franz Duca, Christian Ebner, Marcus Hacker, Renate Kain, Andreas Kammerlander, Matthias Koschutnik, Alexander Stephan Kroiss, Agnes Mayr, Christian Nitsche, Peter P. Rainer, Susanne Reiter-Malmqvist, Matthias Schneider, Roland Schwarz, Nicolas Verheyen, Thomas Weber, Marc Michael Zaruba, Roza Badr Eslam, Martin Hülsmann, Julia Mascherbauer

**Affiliations:** 1 Department of Internal Medicine II, Division of Cardiology, Medical University of Vienna, Vienna, Austria; 2 Department of Medicine III (Cardiology and Angiology), Medical University of Innsbruck, Innsbruck, Austria; 3 Division of Cardiology, Medical University of Graz, Graz, Austria; 4 Department of Medicine I, Division of Hematology & Hemostaseology, Medical University of Vienna, Vienna, Austria; 5 Department of Orthopedics and Trauma Surgery, Medical University of Vienna, Vienna, Austria; 6 Internal Department II of Cardiology, Angiology and Internal Intensive Medicine, Elisabethinen Hospital, Linz, Austria; 7 Division of Nuclear Medicine, Department of Biomedical Imaging and Image-guided Therapy, Department of Radiology and Nuclear Medicine, Medical University of Vienna, Vienna, Austria; 8 Department of Pathology, Medical University of Vienna, Vienna, Austria; 9 Department of Nuclear Medicine, Medical University of Innsbruck, Innsbruck, Austria; 10 Department of Radiology, Medical University of Innsbruck, Innsbruck, Austria; 11 Department of Cardiology, Kaiser Franz Josef Hospital, Clinic Favoriten, Vienna, Austria; 12 Specialist in Internal Medicine and Cardiology, Ried im Innkreis, Austria; 13 Department of Internal Medicine 2 (Cardiology & Intensive Care), University Teaching Hospital Klinikum Wels-Grieskirchen, Wels, Austria

**Keywords:** Cardiomyopathy, Heart failure, 99mTc-DPD scan, Cardiac MRI, Biopsy

## Abstract

The prevalence and significance of cardiac amyloidosis have been considerably underestimated in the past; however, the number of patients diagnosed with cardiac amyloidosis has increased significantly recently due to growing awareness of the disease, improved diagnostic capabilities and demographic trends. Specific therapies that improve patient prognosis have become available for certain types of cardiac amyloidosis. Thus, the earliest possible referral of patients with suspicion of cardiac amyloidosis to an experienced center is crucial to ensure rapid diagnosis, early initiation of treatment, and structured patient care. This requires intensive collaboration across several disciplines, and between resident physicians and specialized centers. The aim of this consensus statement is to provide guidance for the rapid and efficient diagnosis and treatment of light-chain amyloidosis and transthyretin amyloidosis, which are the most common forms of cardiac amyloidosis.

## Introduction

Amyloidosis is a complex multisystem disease that leads to substantial morbidity and mortality. The disease arises from deposition of misfolded protein fragments in organs, such as the heart, kidneys, bone marrow, gut, and nervous system [1, 2].

In recent years, cardiac amyloidosis has developed from a rare disease that was frequently diagnosed only post-mortem, into a clinically relevant condition that every cardiologist and every physician in general can come across [3]. An autopsy study conducted in Finland showed that cardiac amyloidosis was present in 25% of people aged >85 years [4]. The prevalence of cardiac amyloidosis among patients who underwent transcatheter aortic valve implantation was reported to range from 8% to 16%; in patients with heart failure with preserved ejection fraction (HFpEF) it was 13%, and in patients with aortic stenosis 8% [5–9]. The prevalence in patients with (paradoxical) low-flow, low-gradient aortic stenosis is estimated to be up to 30% [10].

The most important amyloid fragments deposited in the heart are transthyretin (TTR) and immunoglobulin light chains. Amyloid deposition leads to enlargement of the extracellular space, which primarily causes diastolic dysfunction of the left ventricle. Patients who suffer from cardiac amyloidosis develop progressive heart failure that has an unfavorable prognosis [11]. The diagnosis needs to be swiftly established to provide patients with the best possible treatment options. Specific therapies have become available for the treatment of TTR amyloidosis, and these can improve patient prognosis if they are prescribed in a timely manner.

This guide provides a diagnostic algorithm for patients with cardiac amyloidosis that is based on the most recent literature and the clinical experience of the authors. A critical aspect is the interdisciplinary collaboration between cardiologists, hematologists, neurologists, nephrologists, geneticists, radiologists, specialists in nuclear medicine, pathologists, and specialists in further disciplines.

Moreover, this guide discusses the necessity for screening of particular patient groups and defines these eligible groups. Finally, the currently available options for treatment of cardiac amyloidosis are reviewed.

## Epidemiology

The two most common subtypes of amyloidosis are light-chain (AL) amyloidosis and TTR amyloidosis (known as ATTR amyloidosis).

### AL amyloidosis

AL amyloidosis is the most common type of amyloidosis, with a prevalence of ≥0.3 per 100,000 of the general population [12]. Just over half of the patients with AL amyloidosis are male, with an age peak of 60–69 years [13]. In more than 70% the heart is affected, which accounts for the substantial mortality rate of up to 50% per year after the first cardiac decompensation [14]. In England, 0.7 in every 1000 fatalities are due to systemic amyloidosis [15].

### ATTR amyloidosis

ATTR amyloidosis is currently the second most commonly diagnosed type of amyloidosis. It manifests as hereditary ATTR amyloidosis, which was previously known as ATTRm, but is now classified as ATTR variant (ATTRv) amyloidosis (according to the 2018 nomenclature defined by the International Society of Amyloidosis). ATTR amyloidosis also manifests as wild-type ATTR (ATTRwt) amyloidosis.

#### ATTRv amyloidosis

Clusters of ATTRv amyloidosis have been identified in Portugal (i.e., Val50Met), Japan and Sweden. The Val50Met mutation mainly causes neurological changes (hereditary amyloid neuropathy, HAP). A common mutation that specifically affects the heart is Val142Ile, which has been shown to be carried by 3–4% of African Americans, and by 10% of African Americans with heart failure with reduced ejection fraction (HFrEF) [18, 19].

#### ATTRwt amyloidosis

ATTRwt amyloidosis is predominantly a cardiac amyloidosis, although it can also affect the peripheral nervous system and tendon sheaths [16, 17]. ATTRwt amyloidosis is mainly observed in men aged >60 years, although early manifestations have been described [20, 21]. As the previously indicated autopsy study showed cardiac TTR amyloid in one quarter of individuals >85 years old, [4] this implied that the actual prevalence was higher than had been previously thought. ATTRwt amyloidosis appears to account for a significant proportion of cases of HFpEF, mainly in males [5, 22]. Studies conducted over the last years have demonstrated concomitant ATTRwt amyloidosis in a considerable proportion of patients with HFpEF and severe aortic stenosis [6, 9, 23–26]. Accordingly, the diagnostic work-up is of great importance in these patients. Based on the demographic development, increased awareness, and improved diagnosis of this disease, ATTRwt amyloidosis might turn into the most common type of cardiac amyloidosis in the near future. Patients affected by ATTRwt amyloidosis have a better prognosis than those with cardiac AL amyloidosis, with an average survival of 6 years [27].

### Key messages

- The most common types of amyloidosis include:
  - AL amyloidosis
  - ATTR amyloidosis.
- ATTR includes ATTRwt and hereditary ATTRv, which are predominantly found in Portugal, Japan, Sweden, and the USA.
- Cardiac amyloidosis is identified in:
  - 70% of patients with AL amyloidosis
  - almost all patients with ATTRwt
  - at different frequencies in ATTRv depending on the underlying mutation.

## Pathophysiology

Cardiac amyloidosis is caused by deposition of misfolded proteins in the extracellular space of the heart. These amyloid deposits show a beta-sheet structure and can be identified using Congo red dye [28]. Approximately 36 precursor proteins are known to form amyloid deposits [29]. Amyloidogenic proteins occur as partly folded and completely unfolded precursors. Factors such as low pH, increased temperature and oxidation can tip the balance in favor of these misfolded proteins.

### AL amyloidosis

AL amyloidosis arises based on plasma cell dyscrasia or a clonal B‑cell population. The misfolded immunoglobulin light chains are produced by a plasma cell/B-cell clone [30]. As in multiple myeloma and monoclonal gammopathy of undetermined significance, AL amyloidosis belongs to the plasma cell dyscrasias, although it constitutes a distinct entity [8, 31]. Approximately 35% of patients with multiple myeloma develop amyloidosis in the course of the disease, while only 10% of AL amyloidosis patients are diagnosed with multiple myeloma. AL amyloidosis primarily affects the heart, kidneys, bone marrow, skin, and liver. Light-chain amyloid deposits can have direct cytotoxic effects that are mediated by p38 mitogen-activated protein kinases [32].

### ATTR amyloidosis

The depositing of TTR is either due to a mutation in the *TTR* gene, or more frequently, to a degenerative effect that most commonly emerges in men around the age of 70 years (i.e., senile ATTR amyloidosis). TTR is mainly produced in the liver (95%) and is a homotetrameric protein that consists of beta-folded subunits that transport thyroxin and retinol [33]. The crucial step leading to ATTR amyloidosis is the dissociation of the TTR tetramer into monomers, as this is the prerequisite for the formation of amyloid fibrils. These amyloid fibrils then amalgamate into amyloid plaques that eventually deposit in various organs. TTR amyloid can infiltrate every part of the cardiovascular system, including the myocardium, heart valves, conduction system, and coronary arteries [34].

#### ATTRv amyloidosis

The *TTR* gene, which is located on chromosome 18, encodes 127 amino acids [35]. To date, more than 140 mutations of the *TTR* gene that can induce TTR misfolding have been described. The organ manifestation and severity of ATTRv varies to an enormous degree [36]. Although the mutated protein is present in the patient from birth, insufficient proteostasis (i.e., an imbalance between protein synthesis and degradation that leads to amyloid deposition) only occurs in adulthood and depends on the underlying mutation and age-related comorbidities, such as arterial hypertension, renal failure, and other biochemical processes [37]. The Val50Met TTR mutation primarily affects the neurological system (HAP), while Val142Ile is frequently related to cardiac amyloidosis [16]. In recent years, several Austrian families carrying *TTR* mutations have been identified [38]. The most commonly found mutation in Austria, His108Arg, is related to a mixed cardiac and neurological phenotype.

#### ATTRwt amyloidosis

This amyloidosis subtype predominantly affects the heart and the peripheral nervous system. In ATTRwt, it is not known why the normal unmutated (i.e., wild-type) protein aggregates and deposits in the extracellular spaces. An intrinsic tendency toward the formation of amyloid is likely [39]. Extracellular accumulation of unfolded TTR can occur due to age-related posttranslational protein modifications, combined with failure of its proteosomal clearance [40, 41].

### Key messages

- (Cardiac) amyloidosis is caused by accumulation of misfolded protein (i.e., amyloid) in the extracellular spaces of the heart.
- AL amyloidosis is caused by the depositing of misfolded immunoglobulin light chains, which are produced by a plasma cell clone.
- ATTR amyloidosis is based on the deposition of TTR, which is due to hereditary (ATTRv amyloidosis) or acquired (senile or wild-type; ATTRwt amyloidosis) changes to TTR.
- Hereditary cardiac ATTRv amyloidosis is based on mutations in the *TTR* gene that results in misfolded TTR protein. ATTRv amyloidosis commonly emerges considerably earlier than ATTRwt amyloidosis.
- The reason why unmutated TTR protein aggregates and deposits in the extracellular space in patients with cardiac ATTRwt amyloidosis has not been identified to date. ATTRwt amyloidosis usually emerges in men around the age of 70 years.

## Clinical presentation

Patients with cardiac amyloidosis usually display symptoms indicative of congestive heart failure, such as pronounced fatigue, weakness, dyspnea, and syncope [42]. Subsequently, ascites, peripheral edema, and pleural and pericardial effusion can occur. Hypotension is a typical sign of cardiac amyloidosis, which is due to impaired ejection fraction and/or peripheral vasomotor dysfunction [43]. Amyloid deposits in the conductive system can result in arrhythmia, including atrial fibrillation and atrioventricular block, and also ventricular tachycardia [44]. In the terminal stages of cardiac amyloidosis, patients die due to progressive heart failure or sudden cardiac death based on tachyarrhythmia, bradyarrhythmia, or electromechanical decoupling [42, 45]. At the time of their first visit to the cardiologist, most patients are already at an advanced disease stage characterized by pronounced amyloid deposits. The clinical presentation and prognosis of patients with cardiac amyloidosis vary substantially, depending on the amyloidosis subtype, as indicated in Table 1.

**Table 1:** Most common subtypes of systemic amyloidosis that can include cardiac involvement

Amyloid subtype	Abbreviation	Precursor protein	Site of production	Organs involved	Clinical signs/predominant symptoms	Age at manifestation	Clinical course
Light chain amyloidosis	AL	Monoclonal light chain	Degenerate plasma cells in bone marrow	Heart, kidneys, liver, gastrointestinal tract, peripheral nervous system, soft tissue	Severe proteinuria; weight loss, constipation; periorbital hematoma; macroglossia; rapidly progressing cardiac amyloidosis (due to light chain toxicity)	Peak at 60–69 years	Cardiac involvement in 70% of patients; high mortality rate of up to 50% per year in patients after first cardiac decompensation
Transthyretin wild-type amyloidosis	ATTRwt	Transthyretin	Liver	Primarily heart and peripheral nervous system	Primarily heart failure; bilateral carpal tunnel syndrome (usually 10 years before cardiac manifestation); rupture of the biceps tendon; spinal canal stenosis	Mainly males >60 years; early manifestations have been described	Average survival 6 years; more favorable prognosis than cardiac AL amyloidosis
Hereditary transthyretin variant amyloidosis	ATTRv	Transthyretin	Liver	Heart and peripheral nervous system, autonomous nervous system, gastrointestinal tract	Clinically heterogeneous, from almost isolated neuropathy to cardiomyopathy overlap syndrome; most commonly peripheral sensorimotor neuropathy with motor impairment and neuropathic pain; gastrointestinal dysautonomia with weight loss and orthostatic hypotension; arrhythmia, syncope, sudden cardiac death	In adulthood due to age-associated comorbidities such as arterial hypertension, renal failure, and other biochemical processes	Organ manifestations and severity vary considerably; in patients with Val50Met, nervous system is mainly affected (familial amyloid polyneuropathy); Val142Ile frequently associated with cardiac amyloidosis

### Signs indicating the cause of cardiac amyloidosis

Certain red flags can be indicative of cardiac amyloidosis, as detailed in Table 2.

**Table 2:** Red flags that indicate cardiac amyloidosis [50]

Reduction in longitudinal strain with apical sparing (cherry on the top)
Discrepancy between left ventricular wall thickness and lack of left ventricular hypertrophy on electrocardiogram
Atrioventricular block with hypertrophic phenotype
Echocardiographic hypertrophic phenotype with associated infiltrative features, including increased thickness of atrioventricular valves, interatrial septum, and right ventricular free wall
Marked extracellular volume expansion and/or diffuse late gadolinium enhancement on cardiac magnetic resonance tomography
Symptoms of polyneuropathy and/or dysautonomia
History of unilateral or bilateral carpal tunnel syndrome
Mild increase in troponin levels on repeated occasions

#### AL amyloidosis

AL amyloidosis is a systemic disease that mainly affects the kidneys, where it can cause severe proteinuria, and the heart. Also, the liver, the peripheral nervous system, and the gastrointestinal tract can be involved, and weight loss and constipation can occur [46]. Periorbital hematoma (i.e., raccoon eyes) due to increased permeability of the capillaries, as well as macroglossia (10% of cases) are strongly suggestive of amyloidosis and should prompt diagnostic assessment [2].

Amyloid deposition in the heart is generally less pronounced in patients with AL amyloidosis than in those with ATTR amyloidosis; however, cardiac AL amyloidosis is more progressive and shows comparatively higher mortality, which mainly arises from the cardiotoxic effects of the light chains [22, 45].

#### ATTRwt amyloidosis

Patients with ATTRwt most often present with symptoms typical of heart failure. The majority of these patients are men above the age of 60 years, while women only constitute approximately 20% [44]. A common clinical sign is bilateral carpal tunnel syndrome (30–50% of cases), which can precede cardiac manifestation by 10 years [19, 27, 44]. ATTRwt is also associated with increased risk of rupture of the biceps tendon, and with spinal canal stenosis [47].

#### ATTRv amyloidosis

The clinical presentation of ATTRv is heterogeneous and greatly depends on the underlying mutation (i.e., the genotype–phenotype association). It ranges from isolated neuropathy to the cardiomyopathy-overlap phenotype [26, 48]. Most commonly, patients show peripheral sensorimotor neuropathy with motor impairment, which can be as severe as complete immobility and neuropathic pain [49]. Autonomic dysfunction includes gastrointestinal dysautonomia followed by weight loss and orthostatic hypotension. In patients with cardiac involvement, arrhythmia, syncope, and sudden cardiac death can occur in addition to the clinical syndrome of heart failure.

### Key messages

- Patients with cardiac amyloidosis typically present with signs and symptoms of heart failure.
- Certain clinical signs are suggestive of distinct subtypes of cardiac amyloidosis (AL: periorbital hematoma, macroglossia; ATTRwt: carpal tunnel syndrome, rupture of the biceps tendon; ATTRv: peripheral sensorimotor neuropathy with motor impairment and neuropathic pain).
- The clinical picture and prognosis of patients with cardiac amyloidosis vary considerably according to amyloidosis subtype. The relative mortalities are: AL > ATTRv > ATTRwt (Table 1).

## Diagnostic algorithm

**Fig. 1:**
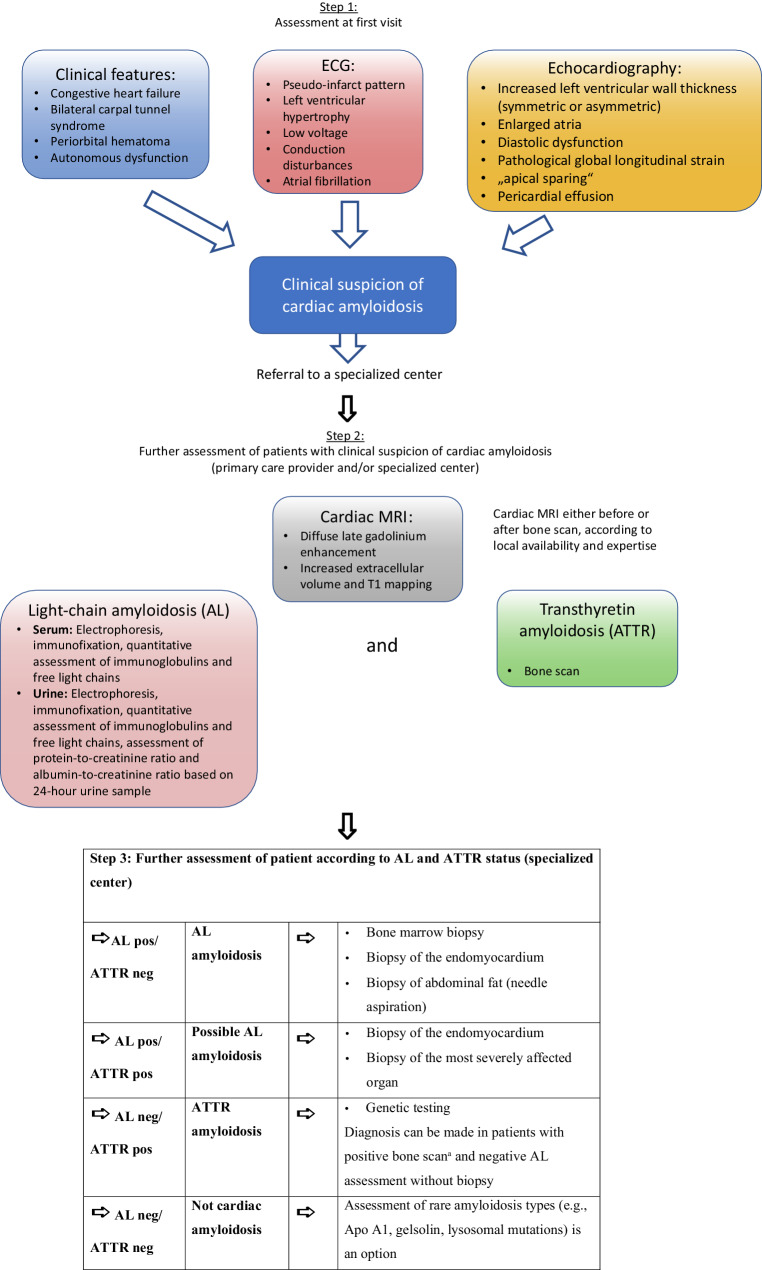
Diagnostic algorithm for cardiac amyloidosis (modified from [16]). ^a^Perugini score 2 or 3. *AL negative* no evidence of free light chains, *AL positive* evidence of free light chains in serum and/or urine, *ATTR negative* bone scan Perugini score ≤1, *ATTR positive* bone scan Perugini score ≥2, *MRI* magnetic resonance imaging, *ECG* electrocardiography

Due to the substantial mortality of patients with AL amyloidosis, rapid diagnostic assessment is necessary if cardiac amyloidosis is suspected. Fig. 1 illustrates a diagnostic algorithm for cardiac amyloidosis that has been shown to work well in clinical practice.

### Step 1 (first visit)

- Clinical assessment/patient history
- 12-lead electrocardiography (ECG)
- Echocardiography
- Cardiac magnetic resonance imaging (CMR)

The diagnosis of suspected cardiac amyloidosis is established based on the clinical picture, ECG, and echocardiography. The red flags that can indicate cardiac amyloidosis are detailed in Table 2 [50]. CMR is helpful to confirm a suspected diagnosis at this time, although access to CMR can be limited, based on restricted local availability and lack of radiologist expertise. In-depth evaluation should be conducted either at a center with experience in the care of patients with cardiac amyloidosis, or locally, depending on the local expertise and the availability of subsequent diagnostic methods, including CMRI.

### Step 2 (primary care provider and/or specialized center)

- Laboratory tests
- Scintigraphy

Based on the findings (i.e., AL-positive/ATTR-negative; AL-positive/ATTR-positive; AL-negative/ATTR-negative; AL-negative/ATTR-positive; see Fig. 1), step 3 might follow.

### Step 3 (specialized center)

- Biopsy of the most affected or most easily accessible organ at a specialized center (this step is mandatory to confirm AL amyloidosis; in patients with ATTR amyloidosis, it is only necessary if the overall picture is not clear).
- Genetic testing (differentiation between ATTRwt and ATTRv).

### Key messages

- In addition to clinical assessment, methods that provide evidence of cardiac amyloidosis include ECG, echocardiography, and CMR, provided that the latter is readily available.
- If the suspicion is substantiated, further evaluation should be carried out at or in close collaboration with a specialized center (i.e., laboratory testing, scintigraphy, biopsy, genetic testing).
- The red flags that can indicate cardiac amyloidosis are summarized in Table 2.

## Diagnostic methods

### Step 1: electrocardiography, echocardiography, and magnetic resonance imaging

#### 12-lead electrocardiography

With ECG, low voltage or lack of signs suggestive of cardiac hypertrophy coinciding with a hypertrophic phenotype indicate cardiac amyloidosis and enable distinction from other hypertrophic cardiomyopathies; however, the diagnosis cannot be made based on the QRS complex morphology alone, as the prevalence of low voltage in patients with cardiac amyloidosis is <50% and as low as 20–35% in those with cardiac ATTR amyloidosis, although these patients show more pronounced amyloid infiltration [51, 52]. Moreover, low voltage is only observed in the later stages of amyloidosis [53]. The increased prevalence of low voltage in AL amyloidosis compared to ATTR amyloidosis might be attributable to increased toxicity of light chains.

In 45–60% of patients with cardiac amyloidosis, the ECG shows a pseudo-infarct pattern (i.e., pathological Q wave in at least two consecutive precordial leads in the absence of coronary artery disease) [44, 46]. Atrial fibrillation and conduction disturbances, such as atrioventricular block or intraventricular block, but also ventricular tachycardia, are common in patients with cardiac amyloidosis [54].

#### Holter ECG monitoring

Disturbances of the stimulation and conduction system are disproportionally frequently observed in patients with cardiac amyloidosis. These can be significant for patient prognosis, as they can give rise to cardioembolic events based on atrial fibrillation, collapse/syncope, or sudden cardiac death due to higher grade atrioventricular block or ventricular tachycardia. In particular, the Austrian hot spot mutation His108Arg is associated with increased incidence of ventricular tachycardia [38].

Holter ECG monitoring at initial presentation and at follow-up (see Table 3) is helpful with respect to the choice of treatment.

**Table 3:** Follow-up assessments in patients with cardiac amyloidosis (modified from the recommendations of the German Cardiac Society [143])

Patient situation	AL amyloidosis			ATTR amyloidosis		
	Frequency (months)	Assessment	Result	Frequency (months)	Assessment	Result
Receiving specific drug treatment	Every 3 (or after every 2 cycles)	NT-proBNP^a^; troponin T or I	Treatment success: >30% decrease; treatment failure, >30% increase; compared to previous result	Every 3‑6	NT-proBNP, troponin T or I	Treatment success: depends on drug^b^
	Every 6	ECG + Holter monitoring; transthoracic echocardiography including strain measurements; if available: heart CMR including LGE and T1 mapping	–	Every 12	ECG + Holter monitoring; transthoracic echocardiography including strain measurements; if available: heart CMR including LGE and T1 mapping	–
After remission or in stable condition who does not receive specific treatment	Every 6	ECG; NT-proBNP^a^; troponin T or I; transthoracic echocardiography including strain measurements	–	Every 6	ECG; NT-proBNP^a^; troponin T or I; transthoracic echocardiography including strain measurements	–
	Every 12	ECG + Holter monitoring; heart CMR including LGE and T1 mapping if laboratory tests or echocardiography suggest progression	–	Every 12	ECG + Holter monitoring; heart CMR including LGE and T1 mapping if laboratory tests or echocardiography suggest progression	–

*CMR* cardiac magnetic resonance imaging, *LGE* late gadolinium enhancement, *ECG* electrocardiogram, *NT-proBNP* N-terminal brain natriuretic peptide

^a^Assessment of NT-proBNP performed when patient is in cardiac compensation, some time after cortisone administration, if applicable

^b^Tafamidis, absence of further NT-proBNP elevations rated as treatment success; patisiran, decrease >30% compared to the last results after 9 and 18 months have been reported [123].

#### Echocardiography

**Fig. 2:**
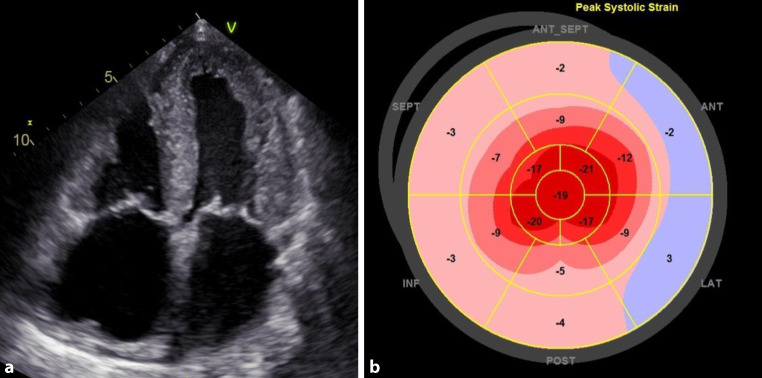
Echocardiographic findings in cardiac transthyretin amyloidosis. **a** Apical four-chamber view showing pronounced thickness of the left and right ventricular walls and the interatrial septum, enlargement of both atria and slight pericardial effusion. **b** Global longitudinal strain analysis in cardiac transthyretin amyloidosis showing apical sparing as a typical sign (i.e., cherry on the top phenomenon). *SEPT* septal, *ANT* anterior, *INF* inferior, *ANT_SEPT* anterior septal, *LAT* lateral, *POST* posterior

Characteristic echocardiographic findings can contribute to rapid diagnosis. Small or regularly sized, hypertrophic ventricles, enlarged atria, and pericardial effusion indicate the possibility of cardiac amyloidosis (Fig. 2a).

Thickening of the left ventricle (LV) wall is due to amyloid infiltration of the extracellular space of the myocardium. The LV mass is measured using M‑mode, 2D, or 3D echocardiography, and should be related to the body surface area [55]. It should be noted that thickening of the LV wall might elude echocardiographic detection in the early stages of the disease or might be within the expected range, e.g., for patients with long-standing hypertension. Another challenge arises from the presence of concomitant high-grade aortic stenosis. Here, it is important not to falsely attribute the thickening of the LV to the valve defect.

Radial LV function is preserved in most patients with cardiac amyloidosis over extended periods, which means that the LV ejection fraction (LVEF) is preserved or only mildly reduced; however, the longitudinal systolic function as assessed by strain analysis (i.e., global longitudinal strain, GLS) is already diminished at the early stages [56, 57]. Therefore, the LVEF to GLS ratio can be helpful to establish diagnosis [58].

A pattern typically found in patients with cardiac amyloidosis consists of preserved apical strain and decreased strain at the base of the heart as well as in the mid-segments of the myocardium [59]. The resulting apical sparing (i.e., cherry on the top) can be visualized using the bull’s eye plot (Fig. 2b; [60]) or the strain ratio. [56–58, 61]. Apical sparing is independent of the extent of wall thickening.

Studies have shown that low GLS [62, 63], low apical longitudinal strain [64] and low basal longitudinal strain [65] are independent predictors of patient survival. In patients with AL amyloidosis, low GLS baseline levels prior to initiation of immunotherapy are predictive of survival [57].

Most patients with cardiac amyloidosis show impaired diastolic function [66]. Signs of diastolic dysfunction can emerge prior to the thickening of the right ventricle (RV) or LV walls, such as atrial dilation and increased E/e′ ratio [67, 68]. Also, the function of the left atrium can be diminished according to atrial strain assessment [67–70]. The commonly described restrictive filling pattern is found in approximately 35% of patients [44].

Furthermore, thickening of the RV wall and impaired RV function are common. Published data indicate that patients with AL amyloidosis and normal LV wall thickness show lower tricuspid annular plane systolic excursion (TAPSE) and lower longitudinal strain of the RV (RV-LS) in the basal lateral segments than control patients, which suggests early systolic dysfunction of the RV [71]. Reduced TAPSE and low RV-LS are also predictive of serious cardiovascular events [63, 68, 72].

Typical signs of cardiac amyloidosis include enlarged atria, thickening of the atrial septum and the valvular leaflets and apparatus, as well as minimal or minor pericardial effusion [51]. Granular sparkling, which has been frequently described, is only seen in 25% of patients with cardiac amyloidosis but also in 12.5% of patients with hypertrophic cardiomyopathy (cave: large interobserver variability) [58].

#### Cardiac magnetic resonance imaging

The relevance and ideal timing of CMR depend to a considerable extent on local accessibility and the expertise of the examining physician. Basically, CMR assessment is already indicated if there is a clinical suspicion in a patient and if echocardiographic findings are suspicious. If this is not the case, specific laboratory evaluation and a bone scan should be preferred.

The advantages of cardiac CMR compared to echocardiography include increased precision in terms of assessment of the structure and function of the heart as well as the possibility of expanded characterization of the myocardium [73, 74]. Moreover, CMR generally allows a differential diagnosis of important conditions, such as hypertrophic cardiomyopathy, or storage diseases such as Fabry’s disease.

**Fig. 3:**
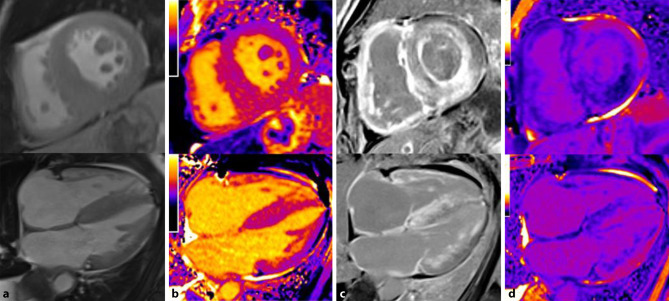
Magnetic resonance tomography in a patient with cardiac transthyretin amyloidosis. Top row: short axis view. Bottom row: four-chamber view. **a** Functional cine imaging showing pronounced left ventricular hypertrophy. **b** T1 mapping showing globally increased T1 relaxation times of 1180 ± 38 ms. **c** Biventricular late gadolinium enhancement with subendocardial enhancement showing characteristic, global patchiness mostly in the basal segments of the myocardium. **d** Extracellular volume (*ECV*): globally increased ECV of 58.4%, estimated according to the formula: ECV = (1 − hematocrit) × [1/post-contrast T1 myocardium − 1/native T1 myocardium]/[1/post-contrast T1 blood − 1/native T1 blood]

At the structural level, ATTR amyloidosis is more commonly characterized by markedly asymmetric LV hypertrophy, while AL amyloidosis often shows less pronounced, symmetric, concentric LV hypertrophy (Fig. 3a); however, the absence of LV hypertrophy does not preclude cardiac amyloidosis [75].

Late gadolinium enhancement (LGE; Fig. 3c) can be used to differentiate cardiac amyloidosis from other pathological changes. In advanced stages of amyloidosis, accumulation of the extracellular contrast agent gadolinium in the expanded interstitial compartment results in the typical diffusely speckled LGE pattern that can be complemented by focal, subendocardial LGE (subendocardial ring). The ATTR amyloidosis subtype often shows transmural LGE, whereas AL amyloidosis frequently demonstrates a subendocardial distribution pattern [76]; however, the enhancement pattern varies considerably, especially in the early stages of the disease; thus, cases of biopsy proven cardiac amyloidosis without pathological LGE have been reported [76].

The newer mapping methods enable quantification of magnetic tissue properties using myocardial T1 relaxation times before and after administration of the contrast agent, which also allows estimation of the extracellular volume (ECV) (Fig. 3b; [77]). While the native T1 time constitutes a composite myocardial signal from myocytes and the interstitium and cannot be used to completely differentiate the underlying disease process (fibrosis, edema, amyloid and/or myocyte necrosis) [78], ECV represents the signal of the extracellular space. A validation study demonstrated that ECV according to CMR correlates well with histological findings based on cardiac biopsies [79].

Increases in both native T1 relaxation time of the myocardium [78, 80, 81] and ECV provide high diagnostic accuracy for cardiac amyloidosis in patients with high pretest probability [82, 83] and enable a prognosis assessment. Overlap with other diseases that also involve LV hypertrophy limit the diagnostic significance of ECV, however [84]. Furthermore, mapping results depend on the device, field strength, and the sequence used, among other aspects, [85] and generally accepted thresholds for ECV and native T relaxation time have not yet been established. Nevertheless, both methods can be used to judge the severity of myocardial involvement and to monitor responses to treatment. [79].

### Key messages

- ECG findings, such as low voltage, pseudo-myocardial infarction pattern, and atrioventricular block, can provide important diagnostic clues for cardiac amyloidosis, although they are not pathognomonic.
- Characteristic echocardiographic results include small or regularly sized, hypertrophic ventricles, markedly enlarged atria, and some pericardial effusion. Also, preserved apical strain and diminished strain at the base are typical. Apical sparing (i.e., cherry on the top) can be visualized using the bull’s eye plot or the strain ratio.
- Cardiac CMR is very helpful to establish a diagnosis of cardiac amyloidosis. The use of CMR in the assessment of amyloidosis or LV hypertrophy of unknown origin depends on the local availability and the expertise of the examining physician.
- New cardiac CMR techniques, such as T1 mapping and assessment of ECV can facilitate the evaluation of disease severity and response to treatment.

### Step 2: laboratory tests and scintigraphy

#### Laboratory tests

The aim of laboratory testing is identification of the cause of amyloidosis, assessment of the affected organs, and risk stratification [86, 87].

##### Pathological light chains

Pathological light chains can be identified using the following tests:

- Serum: electrophoresis, quantitative assessment of immunoglobulins, quantification of free light chains, and immunofixation;
- Urine: electrophoresis, immunofixation, quantification of immunoglobulins and free light chains; protein-to-creatinine ratio and albumin-to-creatinine ratio based on 24‑h urine samples.

##### Troponin (TnT, TnI) and N-terminal brain natriuretic peptide (NT-proBNP)

Both have diagnostic importance, and in addition, they have been established as strong predictors for the clinical course of cardiac amyloidosis [27, 87]. Increased troponin levels are found more frequently in patients with cardiac amyloidosis than in patients with other cardiomyopathies [88]. Both ischemic processes and toxic effects of amyloid might contribute to this finding. The NT-proBNP also appears to be a useful marker for early cardiac involvement [1].

#### Scintigraphy

For patients where there is a suspicion of cardiac amyloidosis, bone scintigraphy is part of the routine evaluation, using tracers such as ^99m^Tc-labeled 3,3-diphosphono‑1,2‑propanodicarboxylic acid (DPD), ^99m^Tc-labeled pyrophosphate, and ^99m^Tc-labeled hydroxymethyl diphosphonate. The exact mechanisms for the increased affinity of these radionuclides toward amyloid deposits in the heart have not been identified yet; however, it has been shown that there is increased, although not exclusive, affinity of radionuclides toward myocardial TTR deposits.

**Fig. 4:**
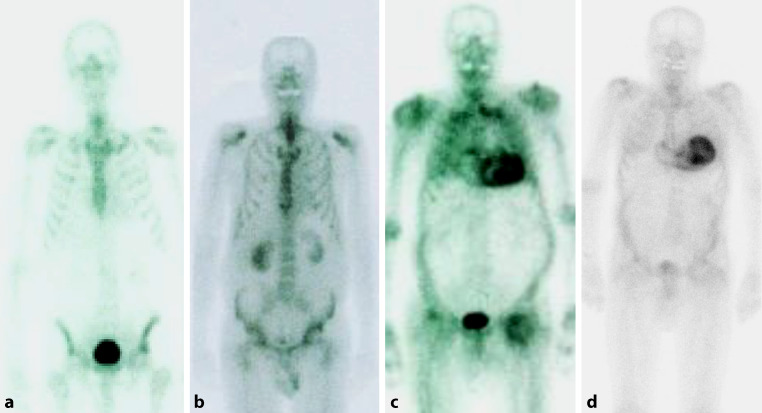
Whole-body scintigraphy using ^99m^Tc-labeled hydroxymethyl diphosphonate. **a** Healthy individual without any cardiac tracer uptake (Perugini grade 0). **b** Patient with cardiac light-chain amyloidosis (AL amyloidosis) and minor cardiac uptake (Perugini grade 1). **c** Patient with cardiac transthyretin amyloidosis (ATTR amyloidosis) and pronounced tracer uptake in the myocardium as well as attenuated osseous uptake (Perugini grade 2). **d** Patient with pronounced tracer uptake in the myocardium but only minor osseous uptake (Perugini grade 3)

Perugini et al. suggested the following grading as the basis of assessment of the radionuclide uptake (Fig. 4; [89]):

- Grade 0: no cardiac uptake, regular osseous structures,
- Grade 1: minor cardiac uptake, with osseous structures appearing comparatively pale,
- Grade 2: moderate cardiac uptake, with osseous structures partly indistinct,
- Grade 3: strong cardiac update, markedly increased extracardiac retention in the soft tissue, with very indistinct osseous structures.

Scintigraphy generally requires interpretation in the context of immunofixation/protein electrophoresis (see above), as pathological findings can also include patients with AL amyloidosis. Radionuclide accumulation does not necessarily correlate with the amyloid burden in biopsic material. [90] The sensitivity of DPD scintigraphy for diagnosis of cardiac ATTR amyloidosis in patients with strong tracer uptake (grade ≥2) has been reported to be up to 100% [31, 89, 91, 92]. In patients with distinct findings (grade ≥2, absence of monoclonal gammopathy), scintigraphy in combination with assessment of free light chains will confirm ATTR amyloidosis. In settings with unclear findings (e.g., grade 1), biopsy is inevitable.

Negative bone scintigraphy does not preclude advanced cardiac AL amyloidosis [31].

#### Positron emission tomography

The evidence available on amyloid positron emission tomography using ^11^C‑Pittsburgh Compound B (PiB), ^18^F‑florbetapir or ^18^F‑florbetaben is limited, so no final conclusion can be drawn at the moment. Basically, this method enables more precise quantification of radionuclide distribution than single photon emission computed tomography. The question of whether this enables differentiation between AL amyloidosis and ATTR amyloidosis cannot be answered at present.

### Key messages

- Laboratory tests enable detection of amyloid precursor proteins (e.g., free light chains) and assessment of organ involvement and patient prognosis.
- Bone scintigraphy is mandatory in the evaluation of patients with suspected cardiac amyloidosis, provided that the diagnosis has not already been established by biopsy.
- Bone scintigraphy findings that are unequivocally positive (i.e., Perugini grade ≥2) are proof of ATTR amyloidosis and preclude AL amyloidosis in the absence of monoclonal gammopathy.

### Step 3: biopsy and genetic testing

#### Biopsy/histology

Histological evidence of amyloid is the diagnostic gold standard for the demonstration of amyloidosis in a patient [31]. With the exception of cardiac ATTR amyloidosis, which can also be diagnosed using a bone scintigraphy, tissue biopsy and subsequent amyloid typing are obligatory for correct diagnosis [91]. Biopsies should be taken from the most severely involved organ after adequate benefit-risk assessment.

The dye of choice for detection of amyloid is Congo red, although it does not allow differentiation of subtypes [93, 94]. This requires immunohistochemistry using monoclonal antibodies against amyloidogenic proteins, and/or mass spectroscopy [31]. It should be noted that a patchy distribution of amyloid within the myocardium can give rise to false negative biopsy findings [95].

Biopsy of the endomyocardium is a relatively safe procedure at experienced centers, with an incidence of life-threatening complications of <1% [96]. In patients where there is a suspicion of AL amyloidosis (i.e., those with typical ECG, echocardiography and/or cardiac CMR and laboratory findings), biopsies from other affected organs are a potential alternative [97, 98]. The organs suitable for noncardiac biopsies include abdominal fat pads obtained by needle aspiration (not skin biopsy), bone marrow, rectum, salivary glands, kidneys, and neural tissue from the lower leg [98]. Of note, negative biopsy findings in other organs do not necessarily preclude cardiac amyloidosis. In such cases, biopsy of the endomyocardium might be necessary after all.

#### Genetic testing

In patients with confirmed ATTR amyloidosis, *TTR* gene sequencing is recommended, as it enables differentiation between ATTRv and ATTRwt. This is important with respect to the range of current treatment options, screening of family members, and improved assessment of prognosis. To date, more than 140 mutations of the *TTR* gene have been described, although not all of these are pathogenic; the pathogenic mutations also vary regarding phenotype and prognosis [99].

Carriers of the amyloidogenic variant often develop the first symptoms only in late adulthood [36]. Presymptomatic genetic testing (i.e., testing of asymptomatic relatives of index patients) can facilitate the treatment of amyloidosis at very early stages and thus possibly improve patient prognosis [100]. Due to incomplete penetrance, genetic counseling is imperative prior to presymptomatic genetic testing [100, 101].

### Key messages

- Cardiac ATTR amyloidosis can be diagnosed based on bone scintigraphy (Perugini score ≥2) and the absence of a monoclonal band in immunofixation/protein electrophoresis. The correct diagnosis of other types of amyloidosis requires evidence of amyloid in tissue biopsies (Congo red staining) and amyloid typing (immunohistochemistry staining, mass spectroscopy).
- Biopsies should be taken from the most severely affected organs after adequate benefit-risk assessment.
- In patients with cardiac ATTR amyloidosis, genetic testing is recommended to distinguish between ATTRwt and ATTRv, as this has prognostic and therapeutic implications. Presymptomatic testing of relatives of index patients with ATTR amyloidosis is reasonable (after genetic counseling) to identify the disease at an early stage or preclude the genetic defect.

## Risk stratification

Troponin T and NT-proBNP are important for the identification of cardiac involvement in amyloidosis. Normal ranges of cardiac biomarkers practically preclude relevant cardiac involvement. If one or more biomarkers are elevated, this can indicate cardiac involvement and can be used for risk stratification in patients with an established diagnosis. The classification systems validated for ATTR amyloidosis by Grogan et al. [21] and Gillmore et al. [102] are based on NT-proBNP plus troponin T, and NT-proBNP plus estimated glomerular filtration rate, respectively. Specific risk stratification procedures for patients with AL amyloidosis are the Mayo Clinic staging system [103] and the revised Mayo Clinic staging system [87]. These are based on troponin T, NT-proBNP and the difference between amyloid-forming and non-involved light chains, respectively. Mortality risk is estimated based on the stages (Table 4).

**Table 4:** Cardiac amyloidosis: risk stratification based on biomarkers

Staging according to:	Amyloidosis	Biomarker				Interpretation	Patient survival	
		NT-proBNP (pg/mL)	Troponin T (ng/L)	FLC-diff. (mg/dL)	eGFR (mL/min)		4‑year survival rates (%)	Mean (months)
Grogan et al. [19]	ATTR	>3000	>0.05	–	–	Stage 1: both parameters < threshold	Stage 1: 57	–
						Stage 2: one parameter > threshold	Stage 2: 42	–
						Stage 3: both parameters > threshold	Stage 3: 18	–
Gillmore et al. [100]	ATTR	>3000	–	–	<45	Stage 1: both parameters < threshold	–	Stage 1: 69
						Stage 2: one parameter > threshold	–	Stage 2: 47
						Stage 3: both parameters > threshold	–	Stage 3: 24
Mayo Clinic staging system [85]	AL	≥1800	≥25.0	≥18	–	1 point/elevated biomarker: stage I–IV	–	Stage I: 94
							–	Stage II: 40
							–	Stage III: 14
							–	Stage IV: 6

Modified from: Yilmaz et al. [143]

*FLC-diff.* difference between free kappa and lambda light chains, *eGFR* estimated glomerular filtration rate

Magnetic resonance tomography can also be useful to determine patient prognosis; however, direct comparisons for the significance of laboratory tests versus that of imaging are missing. Preliminary findings suggest that T1 mapping and ECV calculation provide better risk stratification than laboratory results [78].

### Key messages

- Validated scoring systems have been implemented for risk stratification of patients with ATTR amyloidosis and AL amyloidosis. These are largely based on the biomarkers troponin T and NT-proBNP.
- T1 mapping and calculation of the ECV through CMR can also be used for prognostic assessment.

## Which patients should be screened for cardiac amyloidosis?

### All patients with established or suspected non-cardiac amyloidosis

These include:

- All patients with measurable paraprotein (cave: as pure light-chain paraproteins are not identified by electrophoresis, free light chains should always be tested in serum and urine). Screening should not be restricted to one occasion but should be performed at least once a year throughout the whole course of the disease. AL amyloidosis can develop at any time. It progresses over many months and should be recognized early on, as cardiac amyloidosis is a clear indication for early treatment.
- Patients with established or presumed AL amyloidosis.
- Patients with established or presumed amyloid-related polyneuropathy.
- Patients with established or presumed amyloid-related nephropathy.

### Patients with a hypertrophic cardiac phenotype of unknown origin

These patients include those with symptoms and signs of heart failure, red flags for cardiac amyloidosis (Table 2) and LV hypertrophy (thickness of the myocardium ≥14 mm) that cannot be explained by increased afterload (e.g., hypertension, aortic stenosis). This particularly applies to:

- Older patients (men >65 years, women >70 years),
- Patients with HFpEF and a hypertrophic cardiac phenotype,
- Patients with (paradoxical) low-flow/low-gradient aortic stenosis.

### Key messages

- Screening for cardiac amyloidosis is essential in all patients with established or suspected non-cardiac amyloidosis. Cardiac involvement generally determines the patient prognosis.
- Screening should be conducted in all patients with a hypertrophic cardiac phenotype of unknown origin, particularly in the presence of red flags.

## Treatment options

### Supportive therapies

#### Diuretics

Diuretics have a pivotal role in the treatment of cardiac amyloidosis [102]; however, as patients with cardiac amyloidosis require high intracardiac filling pressure due to increased ventricular stiffness, they are sensitive to hypovolemia. Therefore, diuretics must be used with caution [26, 31].

#### Treatment of heart failure

There is no evidence available in the setting of cardiac amyloidosis that demonstrates benefits of drugs established for heart failure treatment, such as beta-blockers, angiotensin-converting enzyme inhibitors, angiotensin receptor blockers, and angiotensin receptor-neprilysin inhibitors [104]. Indeed, a retrospective study conducted in patients with ATTRv showed decreased survival here [105]. What is more, the tolerability of these drugs is low, as their use can lead to hypotension, and affect the autonomous nervous system. Therefore, in the setting of cardiac amyloidosis, drugs established for heart failure treatment should be avoided whenever possible. This particularly applies to patients with HFpEF. In patients with HFrEF, neurohumoral treatment can be considered if the abovementioned limitations are taken into account.

If heart rate control is called for in patients with tachyarrhythmia, amiodarone and beta-blockers should be preferred over digitalis derivatives and calcium channel blockers. In vitro studies have indicated that these drugs bind to amyloid fibrils, which can increase the local drug levels and might thus give rise to local toxic effects [106].

#### Anticoagulation

Cardiac amyloidosis per se currently has no indication for anticoagulation, although these patients have an increased risk of intracardiac thrombus formation and atrial fibrillation, irrespective of other factors. Thromboembolic events contribute considerably to the increased mortality rate [107].

Even patients with sinus rhythm might develop thrombi of the left atrial appendage based on increased filling pressures, atrial dysfunction, and electromechanical dissociation. Therefore, exclusion of cardiac thrombi via transesophageal echocardiography should be considered prior to cardioversion, even in patients who receive anticoagulation [108, 109]. Accordingly, atrial fibrillation and an intracardiac thrombus are indications for anticoagulation irrespective of the CHA_2_DS_2_-VASC score [110]. In patients with low-amplitude or missing A waves in the transmitral inflow (pulsed wave Doppler; pronounced restrictive filling pattern), anticoagulation can be considered [110].

It should be noted that the risk of hemorrhage can be increased in patients with systemic amyloidosis due to vascular infiltration (i.e., amyloid angiopathy). Also, AL and ATTRv amyloidosis can be accompanied by factor X deficiency, which likewise enhances the bleeding tendency [111]. The clinical significance of potential interactions with factor Xa inhibitors is still not clear.

For selection of anticoagulant therapy, the European guidelines can be followed. The benefits and risks of treatment should be considered in each case, which will be dependent on the individual patient risk of embolic and hemorrhagic events. It is recommended to use direct oral anticoagulants over vitamin K antagonists in the absence of contraindications [112].

#### Devices and ablation therapy

Atrial and ventricular arrhythmias and conduction disturbances are common in patients with cardiac amyloidosis [113]. They are poorly tolerated and often induce clinical deterioration. Electrophysiological studies have shown that atrioventricular conduction disturbances that involve the His Purkinje system are considerably more prevalent than isolated sinus node diseases. Patients with ATTR amyloidosis are more frequently affected by these conditions than those with AL amyloidosis [54, 114, 115]. Accordingly, pacemaker implantation is not uncommon, particularly in the setting of cardiac ATTR amyloidosis [116]. Pacemaker treatment is indicated in patients with a history of syncope or presyncope of unclear origin. Implantation of a loop recorder can be helpful if it is not possible to establish arrhythmia as the cause of these events [117]. If there is an indication for pacemaker treatment in a patient for whom a high right ventricular stimulation fraction is anticipated or who already shows reduced ventricular function, implantation of a cardiac resynchronization therapy (CRT) system or HIS bundle pacing appears reasonable [118]. This can prevent further deterioration of the LVEF, the NYHA class, and mitral valve regurgitation [119]. Basically, the general recommendations for pacemaker and CRT also apply to patients with cardiac amyloidosis [120].

Evidence from trials that have evaluated implantable cardioverter defibrillator (ICD) therapy is scarce and partly divergent. To date, their use as either primary or secondary prevention has not shown distinct reductions in mortality despite high rates of appropriate shock deliverance [115–119].

The European Society of Cardiology (ESC) 2015 guidelines for management of ventricular arrhythmias and prevention of sudden cardiac death recommend implantation of a cardioverter defibrillator as a secondary preventive measure in patients with cardiac amyloidosis who show sustained ventricular arrhythmia (IIA/C recommendation) [120]. The US guidelines recommend individualized treatment selection.

Limited evidence has been generated with respect to catheter ablation in the setting of atrial arrhythmias. Overall, patients with cardiac amyloidosis show significantly higher relapse rates after catheter ablation compared to control patients [121]. A recently published retrospective study in patients with ATTR amyloidosis revealed reduction in mortality and markedly higher efficacy of catheter ablation for atrial fibrillation if this intervention was performed at an early disease stage and patients had less pronounced symptoms (NYHA I/II) [122].

Alternatively, an ablate-and-pace strategy can be used if adequate rate control is not achieved in patients with atrial arrhythmia; however, in this case, the implantation of a CRT system should be preferred.

### Key messages for supportive therapies

- Diuretics are a pillar of supportive treatment; however, dosing requires caution.
- Standard therapies established for heart failure have not been shown to provide distinct benefits for patients with cardiac amyloidosis, and their tolerability is low. These include beta-blockers, angiotensin-converting enzyme inhibitors, angiotensin receptor blockers, and angiotensin receptor-neprilysin inhibitors; however, these drugs can be considered in the setting of HFrEF.
- Anticoagulation therapy should be readily initiated after benefit-risk assessment.
- Conventional pacemaker therapy and cardiac resynchronization are helpful if indicated.
- Evidence supporting implantation of a cardioverter defibrillator as a primary prevention measure is lacking. For secondary prevention, the current ESC guidelines should be followed.

### Amyloid-specific therapies

#### ATTR amyloidosis

Several agents that either stabilize the TTR tetramer or suppress TTR hepatic synthesis have been developed for specific treatment of ATTR amyloidosis. To date, three pharmaceutical products have been approved for the treatment of ATTRv polyneuropathy. Tafamidis (Vyndaqel®) was approved for the treatment of patients with cardiac ATTRv or ATTRwt in the European Union in April 2020.

##### Transthyretin stabilization

The TTR stabilizer tafamidis was licensed in Europe for the treatment of stage 1 amyloid polyneuropathy in 2011 [123]. At present, this is the only drug that has been specifically assessed in patients with cardiac amyloidosis [124]. A recently published phase III study that compared tafamidis 20 mg with tafamidis 80 mg and placebo for 30 months in patients with symptomatic cardiac amyloidosis yielded reductions with tafamidis versus placebo with respect to overall mortality, cardiovascular mortality, and hospitalization rates due to cardiac decompensation. The study population included 106 and 335 patients with ATTRv and ATTRwt, respectively. In addition, improvements were seen for the 6‑min walk test and quality of life using the KCCQ-OS questionnaire [125]. Tafamidis treatment was not associated with serious side effects [126]. Subgroup analyses (i.e., ATTRv vs. ATTRwt; NYHA I and II vs. NYHA III) showed no significant differences in terms of mortality reduction. Interestingly, patients in lower NYHA stages experienced numerically more pronounced improvement in mortality. Hospitalizations due to cardiovascular events occurred less frequently with tafamidis than with placebo in patients with NYHA I and NYHA II, while they were more frequent in those with NYHA III. The authors noted that this might be due to prolonged survival of patients in advanced stages [127]. The available findings support early use of tafamidis.

Likewise, the nonsteroidal anti-inflammatory drug diflunisal was shown to stabilize TTR, although this treatment can result in severe kidney damage [128–130]. Diflunisal is not available in Austria.

##### Inhibition of synthesis of the amyloid precursor protein

In 2018, two drugs were approved in Europe that interfere with TTR-mRNA: the small interfering RNA (siRNA) patisiran (Onpattro®), [131] and the antisense oligonucleotide inotersen (Tegsedi®) [132]. Both agents have been licensed for the treatment of stage I and II ATTRv polyneuropathy. They act by substantially reducing hepatic TTR synthesis. Despite certain differences across the study populations, the available data imply similar efficacy and tolerability of these agents.

Patisiran improved neuropathy and quality of life in patients with ATTRv. In a subgroup with cardiac involvement, patisiran also led to reductions in NT-proBNP levels and improved LV hypertrophy, as well as LV strain [131]. Moreover, a post hoc analysis of this subgroup showed significant reduction in the combined endpoint that included hospital admission and mortality [133]; however, effects of this treatment in patients with cardiac amyloidosis should be interpreted with caution, as cardiac involvement in this study was restricted to LV hypertrophy and was not confirmed otherwise, neither through biopsy nor via cardiac CMR or scintigraphy. Patisiran is administered intravenously every 3 weeks. As transthyretin is involved in vitamin A metabolism, it is recommended to supplement vitamin A during treatment with a siRNA.

Inotersen has shown favorable effects on neurological symptoms and quality of life in patients with cardiac ATTRv. In patients with cardiomyopathy (e.g., LV hypertrophy, heart failure symptoms), inotersen was safe, prevented progression, and tended to reduce the amyloid burden [132, 134]. Inotersen is administered subcutaneously once a week, via a prefilled syringe. The most common adverse events included fever and/or nausea. The risk of thrombocytopenia requires regular blood count monitoring.

Studies assessing patisiran and inotersen in patients with cardiac ATTR are ongoing (patisiran: NCT03997383 APOLLO‑B; inotersen: NCT03702829).

#### AL amyloidosis

##### General principles

In patients with AL amyloidosis, treatment of the underlying clonal B cell disease or plasma cell dyscrasia of the bone marrow has priority and should be initiated as soon as possible. At present, no approved agent is available for patients with AL amyloidosis. Drugs are being used that are established in the treatment of myeloma and lymphoma. Rapid eradication of the amyloidogenic clone and reduction of the amyloidogenic free light chain levels are essential. The choice of agents, their dosing, and the time of administration must be individualized in each case. Treatment becomes more difficult as the disease advances; therefore, the experience of the physician is a determinant for patient survival. No specific cardiac therapy is available.

##### Inhibition of synthesis of the amyloid precursor protein

Patients with AL amyloidosis are mainly treated with bortezomib/lenalidomide-based immunotherapy regimens to inhibit the synthesis of the amyloid precursor protein. Risk stratification at the time of the selection of treatment includes criteria, such as age, Karnofsky index, and extent and impact of organ involvement (i.e., renal function, Mayo and NYHA stages) [135]. Close monitoring is necessary as the duration of treatment is determined by the patient response. If the patient fails to respond, a treatment switch should be considered for implementation after a few cycles.

In general, patients with cardiac amyloidosis show poor tolerance to standard immunotherapy regimens. The anti-CD38 antibody daratumumab (Darzalex®) is a new option for patients with advanced cardiac involvement. Retrospective trials have demonstrated favorable cardiac activity and tolerability of daratumumab [136]. Further studies with more restrictive designs are ongoing (e.g., NCT03201965).

### Transplantation

#### Heart transplantation

Patients with cardiac amyloidosis who do not have significant involvement of other organs are basically candidates for heart transplantation. In the setting of AL amyloidosis, eradication of the paraprotein prior to heart transplantation is deemed beneficial. At present, heart transplantation combined with subsequent autologous blood stem cell transplantation is promoted. The usefulness of this combination will have to be reassessed in light of new agents that are currently being developed [31].

Encouraging data have been generated with respect to heart transplantation in patients with cardiac ATTRv and ATTRwt [26, 137–139]. The basic restrictions and contraindications relating to organ transplantation must also be applied in this setting.

#### Liver transplantation

Liver transplantation is an option in patients with TTR-HAP, as TTR is mainly produced in the liver [26]; however, in Austria, TTR-HAP is not a relevant disease. Liver transplantation appears to be less successful in patients with mutations other than Val50Met [140]. Pre-existing ATTR amyloid deposits can decrease patient quality of life even after transplantation [26, 141]. Considering the limited treatment success and the advances made in the field of drug treatment, liver transplantation is becoming less important, even in regions where TTR-HAP is endemic.

#### Stem cell transplantation

Autologous stem cell transplantation can be suitable for selected patients with AL amyloidosis, and can prevent further amyloid production by plasma cell clones [142].

### Key messages on specific therapies

- Specific agents have been developed for treatment of ATTR amyloidosis that either stabilize the TTR tetramer or suppress TTR synthesis in the liver. The timely use of tafamidis (Vyndaqel®) can improve prognosis of patients with cardiac amyloidosis.
- There is currently no specific treatment for patients with AL amyloidosis. Rapid treatment of the underlying clonal B‑cell disease or plasma cell dyscrasia of the bone marrow is essential.
- Autologous stem cell transplantation, possibly combined with heart transplantation, is an option in selected patients with AL amyloidosis.
- Heart transplantation can be considered in selected patients with ATTR amyloidosis, while liver transplantation is becoming less important.

## Long-term care

For patients with cardiac amyloidosis, structured long-term care is a necessity. In addition to regular treatment monitoring and modifications, customization of management based on repeated risk stratification is useful. The follow-up of patients with cardiac amyloidosis is based on clinical assessment, NT-proBNP or troponin T (or I) measurements, ECG and imaging, such as echocardiography and cardiac CMR [101, 132]. Assessments of LV wall thickness or LVEF alone are insufficient for the evaluation of treatment success and for risk stratification.

Follow-up assessments recommended by the current guideline for patients with cardiac amyloidosis issued by the German Cardiac Society are summarized in Table 3 [143].

### Key message

- Follow-up assessments performed during long-term care of patients with cardiac amyloidosis are based on clinical evaluation, NT-proBNP or troponin T (or I) levels, ECG, and imaging parameters (Table 4).

## Support group, AIDA registry

The support group Amyloidosis Austria (*Leben mit Amyloidose*; https://www.amyloidosis-austria.at) provides information about current scientific insights and treatment options to patients and their relatives, as well as providing a platform for its exchange. This support group collaborates with physicians, public health institutions, and public and private insurance companies.

The Austrian Interdisciplinary Amyloidosis Registry (*österreichisches interdisziplinäres Amyloidose-Register*, AIDA) is an important instrument for quality assurance. AIDA provides information on implementation and revision of guidelines and their efficacy in regional, national and multinational patient populations, and also assessment of adverse events in large patient populations outside of clinical trials, and pharmacoeconomic analyses under real-life conditions.

## Conclusion

Increased awareness of cardiac amyloidosis, improved diagnostic methods, and the demographic development will lead to growing numbers of patients with cardiac amyloidosis in the years to come. This will pose an increasing challenge for physicians working in cardiological practices and specialized centers. Through promising therapies, cardiac amyloidosis has been transformed from a fatal condition to a treatable disease. Rapid and correct diagnosis is the prerequisite for timely treatment.
